# Burnout in Specialized Care Nurses during the First COVID-19 Outbreak in Spain

**DOI:** 10.3390/healthcare10071282

**Published:** 2022-07-11

**Authors:** María Dolores Ruiz-Fernández, Cristina Alarcón-Ortega, María Isabel Ventura-Miranda, Ángela María Ortega-Galán, Andrea Alcaráz-Córdoba, Antonia Berenguel-Marínez, María Jesús Lirola-Manzano

**Affiliations:** 1 Department of Nursing Science, Physiotherapy and Medicine, University of Almería, 04120 Almería, Spain; mrf757@ual.es (M.D.R.-F.); andrea.alco16@gmail.com (A.A.-C.); 2 Facultad de Ciencias de la Salud, Universidad Autónoma de Chile, Temuco 4780000, Chile; 3 Andalucian Health Service, Av. de la Constitucion 18, 41071 Sevilla, Spain; cristinaalarcon90@hotmail.com (C.A.-O.); antoniaberenguelmartinez@gmail.com (A.B.-M.); 4 Department of Nursing, University of Huelva, 21071 Huelva, Spain; angela.ortega@denf.uhu.es; 5 Department of Psychology, University of Almería, 04120 Almería, Spain; mariajesus.lirola@ual.es

**Keywords:** nurses, burnout, COVID-19, atención especializada, variables sociolaborales

## Abstract

*Background:* One of the most outstanding consequences of the pandemic is the impact it had on the mental health of nurses caring for patients with COVID-19 in specialised services. *Aim:* The aim was to analyse the burnout levels of nursing professionals during the COVID-19 pandemic in specialised care and their relationship with variables of the socio-occupational context. *Method*: This was a quantitative, descriptive, observational, cross-sectional study, which included a sample of 355 nursing professionals. The instrument used was a questionnaire (Maslach Burnout Inventory Human Services Survey (MBI-HSS)). *Results*: A mean score of 27.44 (*SD* = 12.01) was obtained in the subscale “Emotional exhaustion”; in “Depersonalisation”, the mean score was 7.26 (*SD* = 6.00); and, finally, in “Personal fulfilment”, the mean score was 38.27 (*SD* = 7.04). Statistically significant differences were found in the subscale “Emotional exhaustion”, which is higher in women than in men. The subscale “Personal Accomplishment” was higher in the age group 51–65 years. Regarding the “Depersonalisation” subscale, statistically significant differences were found with respect to the years of experience in the current service, which is higher in the group aged 39 years or more. *Conclusion*: Intervention programmes are required in healthcare systems to improve the emotional well-being of nursing professionals.

## 1. Introduction

The 2019 coronavirus disease pandemic (COVID-19) has become the most important health crisis of the current era [1]. This health crisis has highlighted the essential role of healthcare professionals as a key element in its containment [2]. Providing nursing care in this context can have important short- and long-term consequences for nursing professionals [3]. One of the most prominent consequences of the pandemic is the impact it has had on the mental health of these professionals caring for patients with COVID-19 [4]. The COVID-19 pandemic emergency has created the need for an organisational renewal of care pathways based on the principles of “primary health care” recommended by the World Health Organization (WHO) [5]. The negative consequences in the work context can cause these professionals to suffer physical and psychological exhaustion [6] along with professional burnout, negatively affecting their health and job satisfaction [7]. They may even suffer from certain syndromes, such as burnout [8]. According to the International Classification of Diseases (ICD-11), burnout syndrome or “occupational burnout” is a syndrome conceptualised as a result of chronic stress in the workplace that has not been successfully managed. It is characterised by three dimensions: (1) feelings of lack of energy or emotional exhaustion; (2) increased mental distance from work, negative or cynical feelings about work or depersonalisation; and (3) reduced professional efficacy or self-fulfillment [9]. Nursing professionals who suffer from this syndrome are often more likely to make mistakes, have reduced job performance, or have lower job satisfaction [10]. Burnout has been related to certain socio-demographic factors, such as gender and age [11], and it was also found in previous research that certain variables in the work context produce higher levels of burnout and, therefore, emotional discomfort triggering psychiatric pathology [12]. Working in emergency and intensive care services has been associated with a greater susceptibility to developing psychiatric disorders [13]. Even seniority in the service is more likely to present with clinical anxiety [14].

During the COVID-19 pandemic, nurses were categorised as vulnerable staff due to fear of infection, high workloads, high patient-to-nurse ratios, long shifts, and not obtaining sufficient personal protective equipment. Nurses who provided care in specific COVID-19 services during the pandemic reported a high prevalence of burnout [15,16]. Shoja [17] found that the levels of job stress and burnout in the group of nurses who were exposed to COVID-19 patients were higher than in the group of nurses who did not work with exposure to COVID-19 patients. However, there is insufficient evidence to determine the prevalence of burnout among different specialised care services during the pandemic [18]. Therefore, the aim of this research was to analyse the levels of burnout in specialised care nurses during the COVID-19 pandemic in relation to a series of socio-demographic and work context variables.

## 2. Materials and Methods

### 2.1. Desing

A quantitative, cross-sectional, descriptive observational study was carried out.

### 2.2. Participants and Setting

A sample size adequate for inference was calculated with a margin of error of 5% and a confidence level of 95%. The estimate for a population of 3071 nursing professionals was 343 nurses who needed to answer the question with adequate reliability. Given the response rate in similar studies in this health care group, it was not necessary to estimate for losses as the calculated size was feasible to obtain. The study included a sample of 355 nursing professionals who were serving in specialised care in two hospitals in the province of Almería, southeastern Spain: Hospital Universitario Torrecárdenas (n = 190) and Hospital de Poniente (n = 165) during the first outbreak of COVID-19 (April–May 2021). Study participants were selected by non-probabilistic purposive sampling [19]. The inclusion criteria were nursing professionals actively providing services in specialised care during the COVID-19 pandemic. The exclusion criteria were all healthcare professionals who were not actively providing services during the pandemic and who refused to participate in the study.

### 2.3. Instrument

The Maslach Burnout Inventory Human Services Survey (MBI-HSS) [20] was used, adding a series of socio-demographic variables: gender, age, place of work, years of experience in the current service, and years of professional experience. This questionnaire has shown good behavior and fit to the data in the factor analysis, and most authors consider that it is a questionnaire with good internal consistency and can be considered valid and reliable in its Spanish version [21]; in this study, it showed a reliability of 0.72.

The questionnaire consists of 22 items in the form of statements about the feelings and attitudes of the professional at work. It measures the 3 aspects of burnout syndrome: (1). The Emotional Exhaustion subscale, which assesses the experience of being emotionally exhausted by the demands of the job. It consists of 9 items (1, 2, 3, 6, 8, 13, 14, 16, 20) and has a maximum score of 54; (2). The Depersonalisation subscale, which assesses the degree to which one recognises attitudes of coldness and aloofness. It consists of 5 items (5, 10, 11, 15, 22) and a maximum score of 30; (3). The Self-fulfillment subscale, which assesses feelings of self-efficacy and self-fulfillment at work. It is composed of 8 items (4, 7, 9, 12, 17, 18, 19, 21) and a maximum score of 48. The items were answered by workers using a Likert-type frequency scale ranging from zero “0” (Never) to “6” (Daily) [22]. High scores on the first two subscales (Emotional Exhaustion and Desperonalisation) and low scores on the third one (Personal Accomplishment) define burnout syndrome.

### 2.4. Data Collection

The questionnaire was digitalised using Google Forms and sent by WhatsApp messaging application to the specialised care nursing staff of the hospitals in the province of Almería during the months of April and May 2021. This application was used due to the confinement caused by COVID-19. In the digitalisation of the questionnaire, the introduction of any personal data was avoided, which allowed anonymisation and lack of traceability at all times, making it a totally anonymous tool for the researcher receiving the data. The estimated time for answering the questionnaire was 10 min. Participation was strictly voluntary, anonymous, and confidential.

### 2.5. Data Analysis

An analysis was carried out using the SPSS statistical software (IBM Corp, Armonk, NY, USA), version 26.0, which gave us the necessary information to express the results obtained. Quantitative variables were calculated using measures of central tendency (mean) and dispersion (standard deviation, minima, and maxima), while percentages and frequencies were calculated for qualitative variables. The Kolmogorov–Smirnov test was performed to check the normality of the quantitative variables. The Student’s t-test and the ANOVA test were used for the comparison of independent variables (sociocollaborative variables) with respect to the dependent variable (total score and subscales of the MBI-HSS questionnaire). The post hoc Significant Mean Difference (SMD) test was used to establish between which categories the significant differences occurred. The confidence level of the study was 95%, and the effect size was analysed with Cohen’s d (d) (small = 0.2; medium = 0.5; large = 0.8) and eta squared (η2) (small = 0.01; medium = 0.06; large = 0.14) on the significant variables (reference).

### 2.6. Ethical Issues

The research project was approved by the provincial Research Ethics Committee of Almeria with internal code 41/2021. The study was carried out according to the standards of appropriate clinical practice and following the international and national standards that regulate Biomedical Research, especially the Declaration of Helsinki and Law 14/2007 of 3 July on Biomedical Research, as well as Regulation (EU) 2016/679 of the European Parliament and of the Council of 27 April 2016 and Law 3/2018 of 5 December on the Protection of Personal Data and Guarantee of Digital Rights. The confidentiality of the data provided by the participants was guaranteed in accordance with Organic Law 15/1999, of 13 December, on the Protection of Personal Data.

## 3. Results

### 3.1. Socio-demographic and Laboral Variables of Health Professionals

A total of 355 healthcare professionals participated with a mean age of 27.44 years (*SD* = 12.02). A total of 41.7% of the participants were in the range of 36 to 50 years old. In terms of gender, 19.89% were male and 83.1% female, with a working experience of 6 to 16 years (39.2%). A total of 36.3% worked in the emergency department, followed by 18.6% who worked in COVID hospitalisation. Finally, 51.5% (n = 183) had less than 5 years of experience in their current service, followed by 31.3% (n = 111) with 6 to 16 years (Table 1).

### 3.2. Burnout and Demographic and Occupational Variables

Table 2 shows the results obtained for each of the subscales of the questionnaire. In the “Emotional Exhaustion” subscale, 52.7% of the participants presented a high level, 46.2% a low level in the “Depersonalisation” subscale, and 49.6% presented high levels in the “Self-realisation” subscale.

When analysing the three subscales of the questionnaire, a mean score of 27.44 (*SD* = 12.01) was obtained in relation to the subscale “Emotional exhaustion”; in the subscale “Depersonalisation”, the mean score was 7.26 (*SD* = 6.00); and, finally, in the subscale “Self-realisation”, the mean score was 38.27 (*SD* = 7.04). The mean total score of the questionnaire was 72.97 (*SD* = 14.51). The subscales of the MBI-HSS questionnaire were related to gender, age, place of work, years of professional experience, and years of professional experience in current service. In relation to sex, statistically significant differences were found in the MBI-HSS subscale, “Emotional Exhaustion” (*p* = 0.01, *d* = *0*.27), which was higher in women (*M* = 28.04, *SD* = 11.39) than in men (*M* = 24.5, *SD* = 14.45). Regarding the MBI-HSS subscale, “Depersonalisation”, statistically significant differences were found with respect to the years of experience in the current service, which was higher in the group of 39 or more years (*M* = 20.0, *SD* = 2.83, η2= 0.05) with respect to the rest of the groups according to the DMS test. In the subscale “Personal fulfillment”, statistically significant differences were obtained in relation to the age group. In order to establish exactly which groups the differences occurred between, the DMS test was carried out. According to this, the differences were significantly higher in the age group 51–65 years (*M* = 40.27, *SD* = 6.49, η2= 0.02) than in the other age groups (Table 3).

## 4. Discussion

The aim of this study was to analyse the burnout levels of nursing professionals working in the different specialised care services during the COVID-19 pandemic. By analysing the results globally, we observed that the participants in this study showed a high level of “Emotional exhaustion”, a low level of “Depersonalisation”, and a high level of “Personal fulfillment”, so they did not meet the criteria that would indicate burnout (high level of “Emotional exhaustion” and “Depersonalisation” and low “Personal fulfillment”).

However, the mean total score of the burnout questionnaire is high. Comparing these results with those of previous studies in the scientific literature shows the existence of high levels of stress and burnout in nursing professionals during the COVID-19 pandemic [23]. At the beginning of the pandemic, there was concern and uncertainty about how the disease would be transmitted [24]. Therefore, the safety of healthcare workers in hospitals is a major concern during the COVID-19 pandemic [25]. An example is a study by Elghazally et al. [26], who observed that during the COVID-19 pandemic, a high prevalence of burnout was recorded among physicians in Egypt. Age, job title, working duration, and working hours/day were significant predictors for burnout syndrome subscale results.

In this study, no signs of burnout were found in nursing professionals working in more stressful services, such as critical care and emergency units. In agreement with other authors [27,28], this could be explained by the empathy that professionals have developed towards patients suffering from COVID-19, a terrifying situation that affects the lives of all people, since, as they refer, a pandemic can trigger compassionate behaviour among nursing professionals, making the nurse-patient relationship more consistent and deeper, and this relationship is beneficial since the treatment and care of patients with COVID-19 improves the morale of these professionals. However, in the study by Bisesti et al. [29], burnout was a common condition among healthcare workers operating in ICUs during the pandemic.

When analysing the results according to the different independent subscales of the MBI-HSS questionnaire, they were found to be in line with other studies [30,31] (both carried out in China) in which the female sex presents a greater risk of “emotional exhaustion” compared to the male sex, due to the fact that women carry a greater number of family responsibilities and burdens [11], so that women bear the fundamental burden of the care system, both formally and informally [32]. However, men may suffer more long-term emotional distress, as men generally experience distress later in life than women [14].

With regard to the years of experience in the current department and “Despersonalisation”, the increase in this subscale is significant, with a greater existence of this subscale in people with many years of work in the same hospital department (39 or more years working). However, according to Galanis et al. [33], a greater risk of burnout in nursing professionals is having little experience working in the same department. This may be due to the fact that repeated exposure to stressful or distressing situations in the same workplace causes empathic distress in nursing professionals. This empathic distress leads to emotional distress or burnout [34].

According to Simonetti’s study [35], older nursing professionals presented a higher risk of suffering burnout syndrome in the “Personal Accomplishment” subscale; however, Galanis et al. [33] associated advanced age and professional experience with greater self-efficacy; reducing symptomatology; and risk of stress, depression, or anxiety. This may be related to the personality of nursing professionals; according to Cañadas-De la Fuente [36], neuroticism, agreeableness, extraversion, and conscientiousness are personality traits that predict at least two of the dimensions of burnout syndrome in nurses. According to Ma et al. [37], leadership reduces burnout and increases the “psychological safety” of nurses. Additionally, as concluded by Rivas et al. [38], resilient nurses were able to cope with stressful situations better. On the other hand, in the study by Socaciu et al. [39], they found no relationship between socio-demographic variables and burnout in nurses, but they did relate it to work and a sedentary lifestyle.

### Limitations and Future Lines

This research may have a number of limitations. Firstly, the majority of the sample was female, which may have biased the results towards this gender. However, we have to bear in mind that this is a women’s profession, so we are really expressing the reality experienced during the pandemic. Secondly, we dealt with questionnaires that were disseminated on social networks, and the bias of social desirability cannot be ignored. In spite of this, we tried to control as much as possible all the variables that could have contributed to it. Finally, this is a descriptive observational study. In this study, it is not possible to determine the causality of certain factors, although it is important because it gives us a description of the situation experienced during the pandemic. There may be certain variables that were not included in the study that may have influenced the burnout of specialised nurses. It is, therefore, necessary that future lines of research continue to delve deeper into the phenomenon of burnout in hospital nurses during the pandemic and establish which variables may have an influence.

## 5. Conclusions

The current COVID-19 pandemic and the resulting health crisis highlighted the attrition of nursing professionals in specialised care services. However, it is true that no significant differences were found between the different specialised services. Socio-demographic variables such as gender and age are related to burnout levels in nursing professionals. The length of experience in the current service is a factor that influences burnout levels. These data indicate that certain nursing professionals are more susceptible to suffering from emotional exhaustion, which can lead to psychiatric pathology in times of health crises. Therefore, it is necessary to implement programmes and strategies that promote compassion and resilience among health professionals at different levels of health care, providing the necessary resources and thus decisive planning for future outbreaks or global epidemics. Health systems must implement these programmes. This issue should not be focused on the nursing profession as the only one responsible for their mental health but should be supported by health care institutions.

## Figures and Tables

**Table 1:** Socio-demographic and employment characteristics of participants (N = 355).

Variable	*n*	%
**Sex**		
Women	295	83.1
Men	60	16.9
**Age**		
From 20 to 35 years	136	38.3
From 36 to 50 years	148	41.7
From 51 to 65 years	71	20
**Workplace**		
COVID Hospitalisation	66	18.6
Non COVID Hospitalisation	46	13
Emergency	129	36.3
ICU	44	12.4
Resuscitation	22	6.2
Operating Room	24	6.8
Outpatients	24	6.8
**Years of professional experience**		
Under 5 years old	60	16.9
6 to 16 years old	139	39.2
17 to 27 years old	93	26.2
28 to 38 years old	54	15.2
39 and over	9	2.5
**Years of experience in current service**		
Under 5 years old	183	51.5
6 to 16 years old	111	31.3
17 to 27 years old	39	11
28 to 38 years old	20	5.6
39 and over	2	0.6

**Table 2:** Frequencies and percentages of MBI results according to scale.

	Emotional Exhaustion		Depersonalisation		Self-Realisation	
	*n*	%	*n*	%	*n*	%
**Low**	99	27.9	164	46.2	72	20.3
**Medium**	69	19.4	78	22.0	107	30.1
**High**	187	52.7	113	31.8	176	49.6

**Table 3:** Burnout, socio-demographic and occupational variables.

	Emotional Exhaustion		Depersonalisation		Self-Realisation		Total Score	
	Mean (SD)	t/F (*p*)	Mean (SD)	t/F (*p*)	Mean (SD)	t/F (*p*)	Mean (SD)	t/F (*p*)
**Sex**		11.37 (0.01)						
Women	28.04 (11.39)		7.30 (6.05)	1.71 (0.19)	38.23 (6.68)	2.68 (0.10)	73.57 (13.82)	3.62 (0.06)
Men	24.5 (14.45)		7.05 (5.84)		38.43 (8.68)		69.98 (17.36)	
**Age**		1.33 (0.26)						2.04 (0.13)
From 20 to 35 years	26.18 (1.35)		7.01 (5.24)	2.64 (0.07)	37.81 (7.13)	3.64 (0.03)	71.00 (13.54)	
From 36 to 50 years	28.49(77,81)		8.03 (6.72)		37.73 (7.09)		74.25 (14.89)	
From 51 to 65 years	27.69(15.53)		6.11 (5.67)		40.27 (6.49)		74.06 (15.31)	
**Workplace**		1.19 (0.31)						
COVID Hospitalisation	28.85 (13.47)		7.35 (6.00)	0.71 (0.64)	38.67 (7.05)	0.91 (0.49)	74.86 (16.03)	1.48 (0.18)
Non COVID Hospitalisation	26.13 (12.58)		5.83 (5.07)		37.20 (7.95)		69.15 (16.60)	
Emergency	26.69 (11.85)		7.74 (6.34)		38.95 (6.02)		73.39 (14.18)	
ICU	29.43 (11.02)		7.64 (6.14)		36.57 (7.98)		73.64 (12.61)	
Resuscitation	31.14 (12.24)		7.50 (5.65)		39.00 (6.02)		77.64 (13.86)	
Operating Room	24.29 (10.98)		6.33 (5.63)		38.54 (8.06)		69.17 (12.73)	
Outpatients	26.25 (12.02)		7.13 (6.12)		37.71 (8.28)		71.08 (11.99)	
**Years of professional experience**								
Under 5 years old	26.15 (11.92)	1.18 (0.32)	6.73 (5.42)	1.89 (0.11)	39.27 (5.91)	2.19 (0.07)	71.69 (13.62)	1.11 (0.35)
6 to 16 years old	28.64 (12.41)		6.73 (5.06)		37.57 (7.86)		74.07 (15.26)	
17 to 27 years old	29.74 (11.38)		8.42 (7.16)		36.77 (7.74)		75.03 (13.32)	
28 to 38 years old	11.81 (2.64)		6.70 (5.99)		36.00 (9.49)		73.15 (19.72)	
39 and over	28.50 (7.78)		10.11 (8.75)		37.50 (.71)		86.00 (11.31)	
**Years of experience in current service**								
Under 5 years old	26.57 (12.30)	1.01 (0.41)	6.27 (5.09)	4.89 (0.01)	39.32 (7.47)	0.84 (0.50)	72.62 (13.47)	0.98 (0.42)
6 to 16 years old	26.40 (11.53)		7.86 (6.23)		38.36 (6.24)		71.50 (13.07)	
17 to 27 years old	28.56 (11.35)		8.51 (7.07)		37. 54 (7.56)		74.52 (15.73)	
28 to 38 years old	28.33 (13.29)		9.15 (7.94)		38.50 (7.09)		73.54 (14.51)	
39 and over	32.44 (16.04)		20.00 (2.83)		36.00 (10.10)		78.56 (26.45)	

Note = SD = standard deviation; t = Student *t*-test; F = ANOVA; *p* = significance level.

## Data Availability

The data presented in this study are available on request from the corresponding author. The data are not publicly available due to privacy or ethical restrictions.

## References

[1] Rothan H.A., Byrareddy S.N. (2020). The epidemiology and pathogenesis of coronavirus disease (COVID-19) outbreak. J. Autoimmun..

[2] Bueno-Ferrán M., Barrientos-Trigo S. (2021). Cuidar al que cuida: El impacto emocional de la epidemia de coronavirus en las enfermeras y otros profesionales de la salud [Caring for the caregiver: The emotional impact of the coronavirus epidemic on nurses and other health professionals]. Enfer. Clin. (Engl. Ed.).

[3] Fernández R., Lord H., Halcomb E., Moxham L., Middleton R., Alananzeh I., Ellwood L. (2020). Implications for COVID-19: A systematic review of nurses’ experiences of working in acute care hospital settings during a respiratory pandemic. Int. J. Nurs. Stud..

[4] Torres-Muñoz V., Farias-Cortés J.D., Reyes-Vallejo L.A., Guillén-Díaz-Barriga C. (2020). Riesgos y daños en la salud mental del personal sanitario por la atención a pacientes con COVID-19. Rev. Mex. Urol..

[5] Piane M., Bianco L., Mancini R., Fornelli P., Gabriele A., Medici F., Battista C., Greco S., Croce G., Franceschetti L. (2022). Impact of the COVID-19 Pandemic on Clinical Pathways for Non-SARS-CoV-2 Related Diseases in the Lazio Region, Italy. Int. J. Environ. Res. Public Health.

[6] Sorenson C., Bolick B., Wright K., Hamilton R. (2016). Understanding Compassion Fatigue in Healthcare Providers: A Review of Current Literature. J. Nurs. Sch..

[7] Salari N., Khazaie H., Hosseinian-Far A., Khaledi-Paveh B., Kazeminia M., Mohammadi M., Shohaimi S., Daneshkhah A., Eskandari S. (2020). The prevalence of stress, anxiety and depression within front-line healthcare workers caring for COVID-19 patients: A systematic review and meta-regression. Hum. Resour. Health.

[8] Ruiz-Fernández M.D., Pérez-García E., Ortega-Galán N.M. (2020). Quality of Life in Nursing Professionals: Burnout, Fatigue, and Compassion Satisfaction. Int. J. Environ. Res. Public Health.

[9] CIE-11 Estadísticas de Morbilidad y Mortalidad. https://icd.who.int/browse11/l-m/es#/http://id.who.int/icd/entity/129180281.

[10] Hofmeyer A., Taylor R., Kennedy K. (2020). Fostering compassion and reducing burnout: How can health system leaders respond in the COVID-19 pandemic and beyond?. Nurse Educ. Today.

[11] Erquicia J., Valls L., Barja A., Gil S., Miquel J., Leal-Blanquet J., Schmidt C., Checa J., Vega D. (2020). Impacto emocional de la pandemia de COVID-19 en los trabajadores sanitarios de uno de los focos de contagio más importantes de Europa. Med. Clin..

[12] Shaukat N., Ali D.M., Razzak J. (2020). Physical and mental health impacts of COVID-19 on healthcare workers: A scoping review. Int. J. Emerg. Med..

[13] Danet Danet A. (2021). Psychological impact of COVID-19 pandemic in Western frontline healthcare professionals. A systematic review. Med. Clin..

[14] Bahadir-Yilmaz E., Yüksel A. (2020). State anxiety levels of nurses providing care to patients with COVID-19 in Turkey. Perspect. Psychiatr. Care.

[15] Dincer B., Inangil D. (2021). The effect of Emotional Freedom Techniques on nurses’ stress, anxiety, and burnout levels during the COVID-19 pandemic: A randomized controlled trial. Explore (N. Y.).

[16] González-Gil M.T., González-Blázquez C., Parro-Moreno A.I., Pedraz-Marcos A., Palmar-Santos A., Otero-García L., Navarta-Sánchez M.V., Alcolea-Cosín M.T., Argüello-López M.T., Canalejas-Pérez C. (2021). Nurses’ perceptions and demands regarding COVID-19 care delivery in critical care units and hospital emergency services. Intensive Crit. Care Nurs..

[17] Shoja E., Aghamohammadi V., Bazyar H., Moghaddam H.R., Nasiri K., Dashti M., Choupani A., Garaee M., Aliasgharzadeh S., Asgari A. (2020). COVID-19 effects on the workload of Iranian healthcare workers. BMC Public Health.

[18] Gonçalves J.V., Castro L., Rêgo G., Nunes R. (2021). Burnout Determinants among Nurses Working in Palliative Care during the Coronavirus Disease 2019 Pandemic. Int. J. Environ. Res. Public Health.

[19] Fernández-Sola C., Granero-Molina J., Hernández-Padilla J.M. (2020). Comprender Para Cuidar: Avances en Investigación Cualitativa en Ciencias de la Salud.

[20] Maslach C., Jackson E.S. (1981). The measurement of experienced burnout. J. Occupat. Behav..

[21] Olivares V., Gil-Monte P. (2009). Análisis de las Principales Fortalezas y Debilidades del “Maslach Burnout Inventory” (MBI). Cienc. Y Trab..

[22] Olivares-Faúndez V. (2017). Laudatio: Dra. Christina Maslach, Comprendiendo el Burnout. Cienc. Trab..

[23] Batalla-Martín D., Campoverde Espinosa K., Broncano-Bolzoni M. (2020). El impacto en la salud mental de los profesionales sanitarios durante la COVID-19. Rev. Enfermería Y Salud Ment..

[24] Cao W., Fang Z., Hou G., Han M., Xu X., Dong J., Zheng J. (2020). The psychological impact of the COVID-19 epidemic on college students in China. Psychiatry Res..

[25] Bellanti F., Lo Buglio A., Capuano E., Dobrakowski M., Kasperczyk A., Kasperczyk S., Ventriglio A., Vendemiale G. (2021). Factors Related to Nurses’ Burnout during the First Wave of Coronavirus Disease-19 in a University Hospital in Italy. Int. J. Environ. Res. Public Health.

[26] Elghazally S.A., Alkarn A.F., Elkhayat H., Ibrahim A.K., Elkhayat M.R. (2021). Burnout Impact of COVID-19 Pandemic on Health-Care Professionals at Assiut University Hospitals, 2020. Int. J. Environ. Res. Public Health.

[27] Ruiz-Fernández M.D., Ramos-Pichardo J.D., Ibáñez-Masero O., Cabrera-Troya J., Carmona-Rega M.I., Ortega-Galán N.M. (2020). Compassion fatigue, burnout, compassion satisfaction and perceived stress in healthcare professionals during the COVID-19 health crisis in Spain. J. Clin. Nurs..

[28] Guixia L., Hui Z. (2020). A Study on Burnout of Nurses in the Period of COVID-19. J. Psychol. Behav. Sci..

[29] Bisesti A., Mallardo A., Gambazza S., Binda F., Galazzi A., Pazzaglia S., Laquintana D. (2021). Facing COVID-19 Pandemic in a Tertiary Hospital in Milan: Prevalence of Burnout in Nursing Staff Working in Sub-Intensive Care Units. Int. J. Environ. Res. Public Health.

[30] Han L., Wong F.K.Y., She D.L.M., Li S.Y., Yang Y.F., Jiang M.Y., Ruan Y., Su Q., Ma Y., Chung L.Y.F. (2020). Anxiety and Depression of Nurses in a North West Province in China During the Period of Novel Coronavirus Pneumonia Outbreak. J. Nurs. Sch..

[31] Li X., Zhou Y., Xu X. (2021). Factors associated with the psychological well-being among front-line nurses exposed to COVID-2019 in China: A predictive study. J. Nurs. Manag..

[32] Siira E., Rolandsson B., Wijk H., Wolf A. (2020). Beyond the definition of formal care: Informal care arrangements among older swedes who are not family. Health Soc. Care Community.

[33] Galanis P., Vraka I., Fragkou D., Bilali A., Kaitelidou D. (2021). Nurses’ burnout and associated risk factors during the COVID-19 pandemic: A systematic review and meta-analysis. J. Adv. Nurs..

[34] Ortega-Galán Á.M., Pérez-García E., Brito-Pons G., Ramos-Pichardo J.D., Carmona-Rega M.I., Ruiz-Fernández M.D. (2021). Understanding the concept of compassion from the perspectives of nurses. Nurs. Ethics.

[35] Simonetti V., Durante A., Ambrosca R., Arcadi P., Graziano G., Pucciarelli G., Simeone S., Vellone E., Alvaro R., Cicolini G. (2021). Anxiety, sleep disorders and self- efficacy among nurses during COVID-19 pandemic: A large cross-sectional study. J. Clin. Nurs..

[36] Cañadas-De la Fuente G.A., Vargas C., San Luis C., García I., Cañadas G.R., De la Fuente E.I. (2015). Risk factors and prevalence of burnout syndrome in the nursing profession. Int. J. Nurs. Stud..

[37] Ma Y., Faraz N.A., Ahmed F., Iqbal M.K., Saeed U., Mughal M.F., Raza A. (2021). Curbing nurses’ burnout during COVID-19: The roles of servant leadership and psychological safety. J. Nurs. Manag..

[38] Rivas N., López M., Castro M.-J., Luis-Vian S., Fernández-Castro M., Cao M.-J., García S., Velasco-Gonzalez V., Jiménez J.-M. (2021). Analysis of Burnout Syndrome and Resilience in Nurses throughout the COVID-19 Pandemic: A Cross-Sectional Study. Int. J. Environ. Res. Public Health.

[39] Socaciu A.I., Ionut R., Barsan M., Ungur A.P., Rajnoveanu A.G. (2020). Burnout in Gastroenterology Unit Nurses. Int. J. Environ. Res. Public Health.

